# Supplementary material for: Experiences of international medical students enrolled in Chinese medical institutions towards online teaching during the COVID-19 pandemic
Source: PeerJ. 2021 Aug 25;9:e12061. doi: 10.7717/peerj.12061 (PMC8401755; doi:10.7717/peerj.12061)
Supplement: Supplemental Information 2 [file peerj-09-12061-s002.docx]

**Questionnaire**

**Demographic Information**

Gender *

Male

Female

Age *

18-20

21-23

24-26

27 & above

Program (e.g., MBBS, BDS, etc.) *

Your answer

Year of Study *

Year 1

Year 2

Year 3

Year 4

Year 5

Nationality (e.g., Pakistani, Indian, etc.) *

Your answer

Your Current Location? *

Home Country

Other than Home Country

China

**Technology readiness & general information about online classes**

Do you have easy access to the internet? *

Yes

No

If yes, how would you grade your internet connectivity? *

Poor
Fair
Average
Good
Excellent

Do you have an unimpeded electrical supply? *

Yes

No

Does your institution have an online learning management system (LMS)? *

Yes

No

Which of the following device do you use for online classes *

Smartphone
Laptop
Tablet
Desktop
Other

How long before the start of a class are you informed about the lecture schedule? *

One day before
Two days before
Few hours before
One hour before
Other

What is the mode of notification of the class schedule? *

Via email
Via individual text on cell phone
Though social media, e.g., WhatsApp or Facebook
Through an Institution website

Other

When did the online classes start in 2020? *

March

April

May

June

August

What is the duration of online teaching per day? *

One Hour
Two Hours
Three Hours
Four Hours

Other

Are you being assessed at the end of each class through a test or quiz? *

Yes

No

How many subjects are covered in one day? *

1

2

3

4

5 or more

**Please tell us your experiences of online teaching during this COVID-19 pandemic**

*Strongly agree Agree Neutral Disagree Strongly disagree

My institution has an online learning management system (LMS) or Website where all
information about online classes is available

All key information about the course is available on the learning management system (LMS) or Institution Website

All course readings, assignments, and lectures are available online

Students are assisted in overcoming obstacles in accessing the classes or materials

Time allotted for online classes is sufficient

I am able to interact with teachers during online classes

I am able to interact with teachers after online class in Q&A session

Every individual is given a chance to participate and pitch in their ideas during online classes

The teachers are well trained for online classes and are able to use the Video Conferencing App with ease

Attending classes from home hampers my attention and focus

Online classes are equally or more informative as compared to active learning on campus

Online learning fits in my schedules better than normal day to day classes

Demonstration of Practical/clinical/lab work by the instructor during online classes would help me learn in a better way

I would like to have these online sessions continued even after campus classes have started

**Lastly, please tell us the three most crucial improvements required to make online sessions more effective and anything you want to share; please feel free to share**

1-

2-

3-

4-

5-
